# Extended follow-up of a short total diet replacement programme: results of the Doctor Referral of Overweight People to Low Energy total diet replacement Treatment (DROPLET) randomised controlled trial at 3 years

**DOI:** 10.1038/s41366-021-00915-1

**Published:** 2021-07-23

**Authors:** Nerys M. Astbury, Rhiannon M. Edwards, Fitsum Ghebretinsea, Milensu Shanyinde, Jill Mollison, Paul Aveyard, Susan A. Jebb

**Affiliations:** Nuffield Department of Primary Care Health Sciences, University of Oxford, Oxford, UK

**Keywords:** Translational research, Weight management

## Abstract

**Objectives:**

To test the long-term effectiveness of a total diet replacement programme (TDR) for routine treatment of obesity in a primary care setting.

**Methods:**

This study was a pragmatic, two-arm, parallel-group, open-label, individually randomised controlled trial in adults with obesity. The outcomes were change in weight and biomarkers of diabetes and cardiovascular disease risk from baseline to 3 years, analysed as intention-to-treat with mixed effects models.

**Interventions:**

The intervention was TDR for 8 weeks, followed by food-reintroduction over 4 weeks. Behavioural support was provided weekly for 8 weeks, bi-weekly for the next 4 weeks, then monthly for 3 months after which no further support was provided. The usual care (UC) group received dietary advice and behavioural support from a practice nurse for up to 3 months.

**Results:**

Outcome measures were collected from 179 (66%) participants. Compared with baseline, at 3 years the TDR group lost −6.2 kg (SD 9.1) and usual care −2.7 kg (SD 7.7); adjusted mean difference −3.3 kg (95% CI: −5.2, −1.5), *p* < 0.0001. Regain from programme end (6 months) to 3 years was greater in TDR group +8.9 kg (SD 9.4) than UC + 1.2, (SD 9.1); adjusted mean difference +6.9 kg (95% CI 4.2, 9.5) *P* < 0.001. At 3 years TDR led to greater reductions than UC in diastolic blood pressure (mean difference −3.3 mmHg (95% CI:−6.2; −0.4) *P* = 0.024), and systolic blood pressure (mean differences −3.7 mmHg (95% CI: −7.4; 0.1) *P* = 0.057). There was no evidence of differences between groups in the change from baseline to 3 years HbA_1c_ (−1.9 mmol/mol (95% CI: −0.7; 4.5; *P* = 0.15), LDL cholesterol concentrations (0.2 mmol/L (95% CI −0.3, 0.7) *P* = 0.39), cardiovascular risk score (QRISK2) (−0.37 (95% CI −0.96; 0.22); *P* = 0.22).

**Conclusions:**

Treatment of people with obesity with a TDR programme compared with support from a practice nurse leads to greater weight loss which persists to at least 3 years, but there was only evidence of sustained improvements in BP and not in other aspects of cardiometabolic risk.

## Introduction

The results from several international trials investigating the clinical effectiveness of programmes that combine low-energy total diet replacement (TDR) with behavioural support have consistently shown that these TDR programmes are an effective treatment for obesity [1–6]. They promote rapid weight loss over 12–16 weeks and lead to significant and substantial weight loss at 1 year compared with behavioural weight-loss programmes which do not include meal replacements. There is growing interest from healthcare commissioners and policymakers for the potential of TDR as a routine treatment for obesity. The National Health Service (NHS) in England has announced a pilot rollout of this treatment, involving referral of 5000 patients with type 2 diabetes [7, 8] to a 12 week TDR weight loss programme. However, evidence on longer-term outcomes is limited [9].

Unlike most other recent TDR trials [2, 3, 6, 10], the DROPLET study tested the effectiveness of a TDR for routine treatment of obesity, rather than as a specific treatment aimed at diabetes remission. In the DROPLET trial, at 1 year participants in the TDR group lost over 10 kg, 7 kg more than those receiving usual care (UC), with 45% of participants in the TDR group achieving a weight loss of 10% or more [1]. Diastolic blood pressure was 3.1 mmHg lower, with HbA1c reductions of 2.2 mmol/mol compared with UC. The intervention was delivered by referring participants to existing counsellors in the community and economic modelling suggesting that this intervention delivery model was a cost-effective option for treating adults with obesity [11]. This analysis assumed that weight was fully regained over 5 years and that cardiometabolic risk followed a similar pattern. However, this was based on assumptions about weight regain from trials of other weight loss interventions and there is concern that weight regain may be quicker after TDR programmes than other programmes associated with a slower rate of weight loss or those that do not rely on special products [11, 12].

The aim of this study was to determine longer-term weight and health outcomes of the participants of the DROPLET randomised controlled trial. These data will allow healthcare professionals to determine the clinical value of TDR programmes, help in assessing cost-effectiveness, and inform healthcare commissioners.

## Methods

The DROPLET trial was a pragmatic, individually randomised, two-arm, open-label, parallel trial. The participants were randomised in a 1:1 allocation stratified for BMI (<35 kgm^2^ or ≥35 kgm^2^) and type 2 diabetes status to either a TDR treatment or UC (control). A full protocol for the original trial has been published previously [13]. This study was powered to detect a difference in body weight between groups at 1 year (primary outcome) [1]. We obtained additional ethical approval to conduct a 3-year follow-up for this study from NHS NRES South Central Oxford B Research Ethics committee (Ref: 19/SC/0012) and the follow up of the trial was registered with ISRCTN12311645. All participants gave written informed consent before enrolment to the follow-up study.

### Participants and settings

**Fig. 1:**
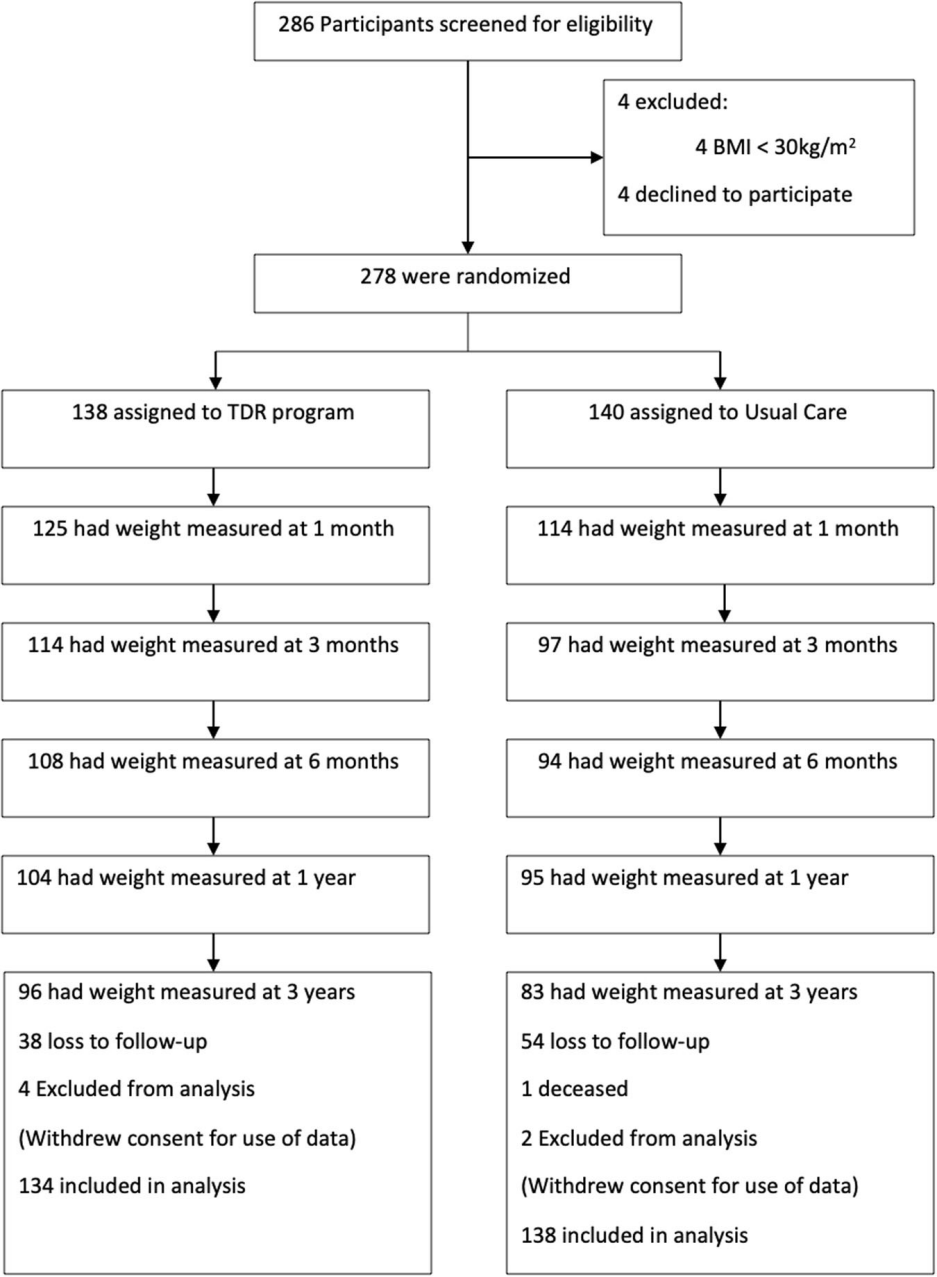
Consort flowchart.

Eligible participants included all those who had taken part in the DROPLET trial, except the few participants who withdrew prior to the 1-year follow-up (Fig. 1). Full trial inclusion and exclusion criteria have been reported previously, but we enrolled adults with obesity, with few exclusions [1, 13]. Eligible participants were re-contacted around the time of the 3 year anniversary of their enrolment on to the DROPLET trial. They were invited to attend an appointment at their GP practice to measure body weight, blood pressure and to provide a fasting blood sample. Participants who were unable to attend follow-up in person were asked if we could use a recent weight and health measures from their medical record for this study. The researchers extracted this information from the participants records if it was available. Where it was not available, participants were asked to provide a self-reported weight measurement. All participants were asked to complete a questionnaire measuring quality of life. Three year follow-ups took place between March 2019 and September 2019.

### Interventions

The TDR programme was provided by Cambridge Weight Plan UK, which manages a network of counsellors providing behavioural support and food products. Participants were asked to contact a local counsellor who delivered a 24-week TDR weight loss programme consisting of 8 weeks TDR, 4 weeks gradual food-reintroduction, and a further 12 weeks weight maintenance. Behavioural support was provided weekly for 8 weeks, bi-weekly for 4 weeks, and monthly for 3 months.

For participants randomised to the UC comparator, practice nurses offered a weight loss programme for 12 weeks, at a frequency typically used in the practice (e.g. weekly or bi-weekly). Full details of the interventions provided as part of the trial have been published previously [13].

Consistent with the pragmatic design, participants in both groups were free to use other weight loss programmes or products during and after their assigned interventions, but GPs were asked not to refer participants to other weight loss programmes for the first 12 months.

### Procedures

At baseline and follow-up, weight and body fat were measured on a digital scale (TANITA SC-240). Waist circumference was measured at the top of the iliac crest [14]. Seated blood pressure was measured three times using an automated blood pressure monitor, with the mean of the last two measurements used for analysis. Measurements were collected by research nurses or a trained research assistant. A fasting blood sample was collected and analysed for glucose, insulin, triglycerides and cholesterol fractions. Participants were asked to complete questionnaires booklet, which recorded quality of life using the EQ5D and obesity-specific OWL-QOL [15–17].

### Outcomes

The primary outcome was change in body weight from baseline to 3 years. The secondary outcomes were: the proportion of participants achieving 5% and 10% weight loss, change in fat mass, LDL cholesterol, HbA1c, systolic and diastolic blood pressure, and 10-year cardiovascular risk using QRISK2 between baseline and 3 years. Although participants were not blinded to treatment allocation, the research nurses and researchers who collected the outcome measures for this 3 year follow-up were blinded to the participants treatment allocation.

Pre-specified exploratory outcomes were change from baseline to 3 years in waist circumference, fasting triglycerides, HDL cholesterol, glucose and insulin, as well as derived HOMA-IR, HOMA-%β, and HOMA-%S calculated using the HOMA2 calculator, and changes EQ-5D and OWL-QOL [15–17]. We also examined weight change from 6 months (end of TDR programme) to 3 years.

### Statistical analysis

We followed a statistical analysis plan finalised prior to database lock. The primary, secondary and exploratory outcomes were all analysed by an independent trial statistician following an intention-to-treat (ITT) analysis in Stata Version 14.0. The primary outcome was analysed using a linear mixed effect model with randomised group, visit, the interaction between visit and randomised group, baseline weight and baseline variables predictive of missing primary outcome (age, gender and diabetes status) included as fixed effects. Practice and participant were included as random effects in the model, to account for repeated measures on the same participant at 3 months, 6 months and 1 and 3 years. An unstructured variance covariance matrix was specified. The analysis of secondary and exploratory outcomes followed the same strategy as the primary outcome with baseline BMI and baseline outcome (if applicable) substituted for baseline weight as a fixed effects. Weight loss of ≤5% and ≤10% at 3 years was analysed using a mixed effect logistic regression model.

We assessed the sensitivity of the primary outcome measure to missing data using different imputation methods; baseline and last observation carried forward, completers only, multiple imputation, and a pattern mixture model assuming different degrees of missing not at random.

To assess whether treatment effects differed by age, gender, BMI, diabetes status and socioeconomic deprivation we performed pre-specified exploratory subgroup analyses.

Participants home postcode were used to calculate patients’ socioeconomic deprivation using the Index of Multiple Deprivation (IMD) [18]. The IMD ranks geographical areas of about 500 households in the UK on seven indices: income, employment, health deprivation and disability, education, crime, barriers to housing and services, and living environment. These ranks are grouped into deciles which were used for analysis with lowest decile [1] representing the most deprived areas and highest decile [10] representing the least deprived areas.

### Patient involvement

All participants in the original trial were sent a summary of the main results and link to access the full manuscript when the results were published in September 2018. Feedback from participants was positive overall, with many commenting that the magnitude of weight loss achieved by the TDR programme was impressive. However, several participants raised concerns regarding the sustainability of the effects. This feedback was integral to our decision to seek support to measure the effects.

## Results

Six of the 278 randomised participants withdrew consent for their data to be used after randomisation, leaving 272 participants eligible for follow-up. At the 3 year follow up we collected outcome data from 179 (66%) participants; 96 (72%) from the TDR group and 83 (62%) from UC group respectively. Body weight was objectively measured in 90 (94%) and 78 (94%) participants in TDR and UC respectively, with the remaining 11(6%) of weight outcomes based on self-reported measures (Fig. 1)

Most participants were middle aged, 55% were women, and 90% were white British. The mean baseline BMI was 36.8 kg/m^2^, 20% were diagnosed with type 2 diabetes and 25% had hypertension at baseline (Table 1).

**Table 1:** Baseline characteristics^a^.

	*N*	Usual care	Total diet replacement
Age (yrs)	179	50.9 ± 11.7	50.7 ± 11.2
Gender n (%)			
Female	99	45 (54.2)	54 (56.3)
Male	80	38 (45.8)	42 (43.8)
Ethnicity			
White British	162	75 (86.2)	87 (90.6)
Not White British	17	4 (23.5)	9 (52.9)
IMD decile^b^	179	7.3 ± 2.0	7.6 ± 2.0
Weight (kg)	179	105.6 ± 18.8	107.4 ± 18.8
Height (cm)	179	169.0 ± 9.6	169.2 ± 9.3
BMI (kg/m^2^)	179	36.1 ± 4.3	37.5 ± 6.0
Waist circumference (cm)	177	114.0 ± 10.5	116.5 ± 13.5
Body fat (%)	172	41.2 ± 7.3	42.5 ± 8.1
Blood pressure (mmHg)			
Systolic	176	130.9 ± 15.2	132.6 ± 15.7
Diastolic	176	80.8 ± 9.9	83.8 ± 9.3
Medical conditions *n* (%)			
Type 2 diabetes	179	20 (14.5)	21 (15.7)
Hypertension	179	30 (21.7)	33 (24.6)
HbA1c (mmol/mol)	176	40.2 ± 12.6	40.7 ± 12.8
Fasting blood glucose (mmol/L)	175	5.8 ± 2.1	6.1 ± 2.7
Fasting insulin (pmol/L)	174	105.3 ± 94.4	95.8 ± 50.6
HOMA -IR	171	1.9 ± 1.5	1.9 ± 1.3
HOMA β (%)	171	129.2 ± 91.6	115.0 ± 56.3
HOMA S (%)	171	69.0 ± 32.1	69.3 ± 36.2
Cholesterol (mmol/L)			
Total	178	5.0 ± 1.1	5.1 ± 1.1
High-density lipoprotein	178	1.2 ± 0.3	1.2 ± 0.3
Low-density lipoprotein	172	3.1 ± 1.0	3.2 ± 0.9
Triglycerides (mmol/L)	178	1.7 ± 0.8	1.7 ± 0.9
QRISK2 (%)	175	9.6 ± 9.0	9.5 ± 8.7

^a^Values represent means ± SD or proportion.

^b^Index of Multiple Deprivation (IMD) ranks geographical areas of about 500 households in the UK on seven indices: income, employment, health deprivation and disability, education, crime, barriers to housing and services and living environment. These ranks are grouped into deciles which were used for analysis with lowest decile representing the most deprived areas and highest decile representing the least deprived area.

### Primary outcome

**Fig. 2:**
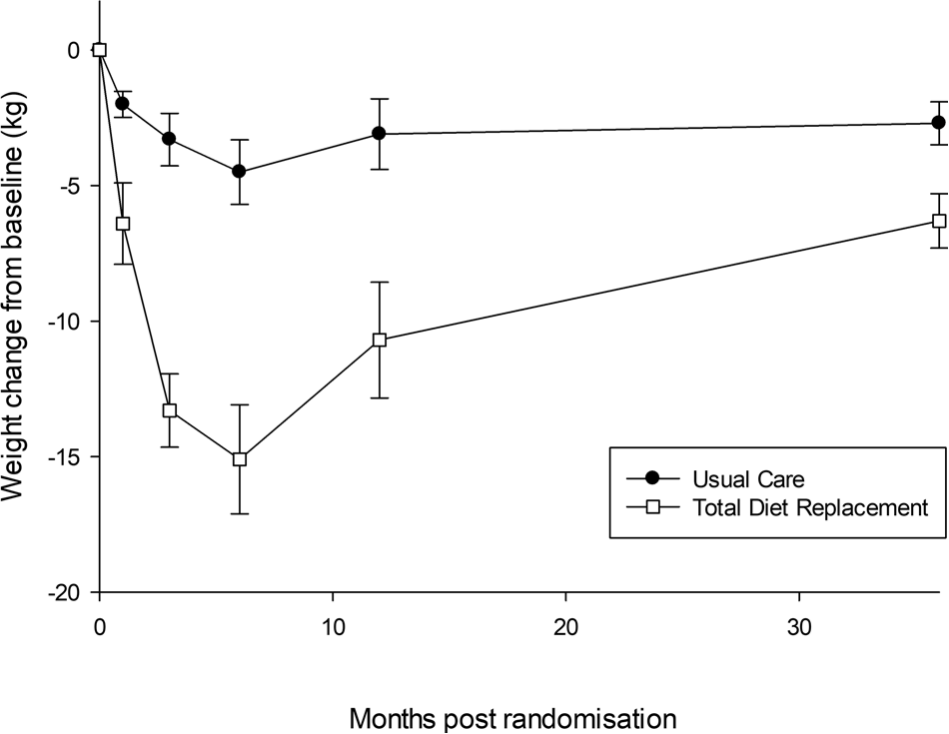
Weight change over 36 months in the intention-to-treat population*. *values represent means ±SEM.

Weight change at 3 years was −6.3 kg (SD 9.1) in the TDR group and −2.7 kg (7.7) in the control group (Fig. 2), adjusted difference −3.3 kg (−9.4; −4.9), *p* < 0.0001. Sensitivity analyses for different assumptions about loss to follow-up did not change the findings (Table S1). In addition sensitivity analysis excluding self-reported outcomes did not change the findings (Table S2).

There was no evidence that the effect of the intervention on weight change differed by age group (*p* = 0.37), baseline BMI (*p* = 0.73) or diabetes status (*p* = 0.35). However, the mean difference between TDR and control groups for weight change from baseline to 3 years was less in women than in men (*p* = 0.039), and there was an indication that participants from more deprived neighbourhoods also received less benefit from TDR than participants from less deprived areas (*p* = 0.058) (Fig. S1).

### Secondary outcomes

At 3 years, 46% and 35% of participants in the TDR and UC group respectively who attended follow-up had lost ≥5% of their baseline weight, odds ratio (OR) 1.5 (0.8; 2.8), *p* = 0.20. Twenty-four percent and 13% of participants lost ≥10% baseline weight in the TDR and UC groups respectively; OR 1.9 (0.8; 4.2), *p* = 0.13 (Fig. S2). The change in fat mass showed similar changes to total body weight, albeit smaller (Table 2).

**Table 2:** Primary, secondary and exploratory outcomes by group.

	Change from baseline (mean ± SD)				Treatment difference	
	Total diet replacement	*n*	Usual care	*n*	Adjusted difference (95% CI)	*p* value
3 years						
Weight (kg)^a^	−6.3 ± 9.1	96	−2.7 ± 7.7	83	−3.3 (−5.2, −1.5)	<0.001
Lost ≥5% weight *n* (%)^b^	44 (45.8)	96	29 (34.9)	83	OR; 1.5 (0.8, 2.8)	0.202
Lost at least ≥10% weight *n* (%)^b^	23 (24.0)	96	11 (13.3)	83	OR; 1.9 (0.8, 4.2)	0.126
Fat mass (kg)^b^	−4.9 ± 10.1	76	−1.9 ± 6.7	69	−2.4 (−4.4; −0.5)	0.016
Systolic Blood Pressure (mmHg)^b^	−4.3 ± 16.4	77	0.0 ± 15.0	68	−3.7 (−7.4; 0.1)	0.057
Diastolic Blood Pressure (mmHg)^b^	−1.2 ± 9.9	77	3.8 ± 10.9	68	−3.3 (−6.2; −0.4)	0.024
HbA1c (mmol/mol)^b^	3.1 ± 9.8	71	−0.8 ± 6.4	61	1.9 (−0.7; 4.5)	0.152
QRISK2 (%)^b^	0.98 ± 2.77	57	1.46 ± 2.15	50	−0.37 (−0.96; 0.22)	0.222
Waist circumference (cm)^c^	−10.5 ± 9.1	77	−5.5 ± 7.3	70	−3.5 (−5.7; −1.4)	0.001
Fasting glucose (mmol/L)^c^	−0.5 ± 1.8	59	0.1 ± 1.3	47	−0.4 (−0.8; −0.1)	0.396
Fasting insulin (pmol/L)^c^	−20.6 ± 35.1	49	−25.3 ± 110.2	44	−1.7 (−17.7; −14.4)	0.839
HOMA- IR^c^	−0.4 ± 0.7	47	−0.5 ± 1.7	41	0.00 (−0.3;−0.3)	0.997
HOMA β (%)^c^	−17.9 ± 35.7	47	−17.1 ± 84.3	41	−4.2 (−17.7; 9.2)	0.537
HOMA S (%)^c^	28.4 ± 58.6	47	20.2 ± 35.2	41	4.9 (−12.7; 22.5)	0.586
Total Cholesterol (mmol/L)^c^	−0.1 ± 0.8	69	−0.2 ± 0.9	61	0.2 (−0.1, 0.4)	0.264
HDL cholesterol (mmol/L)^c^	0.1 ± 0.3	69	0.1 ± 0.2	61	0.00 (−0.1; 0.1)	0.969
LDL Cholesterol (mmol/L)^b^	−0.1 ± 0.7	61	−0.2 ± 0.7	56	0.1 (−0.10; 0.34)	0.295
Triglycerides (mmol/L)^c^	−0.2 ± 0.6	63	−0.1 ± 0.9	58	−0.1 (−0.4; −0.1)	0.394
Quality of Life:						
EQ-5D (Index)^c^	0.00 ± 0.17	67	0.02 ± 0.016	70	−0.02 (−0.07; 0.03)	0.419
EQ-5D (VAS)^c^	6.6 ± 19.9	79	22.6 ± 115.1	70	−18.0 (32.2; 3.8)	0.013
OWL-QOL^c^	9.5 ± 16.2	77	8.1 ± 14.7	70	1.1 (−4.2; 6.5)	0.674

^**a**^Primary outcome.

^**b**^Secondary outcome.

^**c**^Exploratory outcome.

At 3 years, there was no evidence of differences between groups in change in HbA_1c_ or LDL cholesterol. Both systolic and diastolic blood pressure were lower in the TDR group although the between group difference was not significant for systolic blood pressure (Table 2). There was no evidence of a difference in the change in QRISK2 score between groups.

In a post-hoc analysis, we examined the effectiveness of the TDR programme for people with diabetes or hypertension (Table S3 and S4). We advocated reductions in medication for hypertension or diabetes in the initial phase of the TDR programme, which may have masked changes in blood pressure and glucose regulation. In these small subgroups there was no evidence that medication changes differed significantly between groups.

### Exploratory outcomes

#### Changes between end of programme (6 months) and 3 years

Both groups gained weight between 6 months (the end of the programme) and 3 years. The control group gained 1.2 kg (SD 9.1) and the intervention group gained 8.9 kg (SD 9.4), adjusted difference +6.9 kg (95% CI 4.2, 9.5).

The change in waist circumference showed a similar pattern to changes in body weight and fat mass. There was no evidence of differences between groups in glucose regulation, triglycerides or cholesterol fractions between 6 months and 3 years (Table 2).

#### Quality of life

Quality of life measures at 3 years improved in both groups compared with baseline with no evidence of a difference between groups except for the visual analogue scale component of EQ-5D, where the improvement was greater in the UC group (Table 2).

#### Self-reported weight loss attempts

At 3 years, 70% participants in the TDR group and 68% in the UC group who attended follow up reported attempting to lose weight in the 4 weeks prior to their appointment. Of those reporting trying to lose weight, 49% of people in the TDR group, and 50% in the UC group reported that they were attending a formal weight management programme. The mean weight loss at 3 years of the participants reporting trying to lose weight compared with those not currently trying to lose weight was −7.6 kg versus −4.8 kg in the TDR group and −3.6 kg versus −1.6 kg in the UC group.

## Discussion

Participants randomised to a TDR and behavioural support programme for weight loss were on average 6 kg lighter than at baseline, and lost 3 kg more than participants randomised to a UC weight loss programme after 3 years. This is despite greater regain from the end of the programme (weight loss nadir) to 3 years in the TDR group (+8.9 kg) compared with the control (+1.2 kg). Blood pressure was somewhat lower in the TDR group compared with UC, albeit the difference in systolic pressure difference was not significant. There was no clear pattern and no significant differences in measures of glucose regulation. Lipid changes were likewise not significantly different between the groups. Although some people with hypertension and diabetes reduced medication, there is no evidence this accounted for the lack of observed effect of the intervention on cardiovascular risk. Both the main EQ-5D factor score and OWLQOL scales showed improvements in quality of life from baseline but no evidence of differences between groups, although the EQ-5D visual analogue scale showed a significantly greater improvement in quality of life in the UC over the TDR group.

### Strengths and limitations

The major strength of this study is the pragmatic design, which can provide an estimate of the likely outcomes from routine referral systems for the treatment of obesity. Limitations include that the trial was originally planned to assess outcomes at 1 year only. An amendment to the protocol was made to include a follow-up at 3 years, but this was a post-hoc decision after seeing the primary outcome analysis. The lack of contact between the researchers and participants between 1 year and 3 years is likely to have reduced engagement, resulting in one-third of participants not being followed-up. Weight loss at 1 year for the participants who we obtained follow-up measurements at 3 years was greater (TDR −11 kg SD 9.5, UC −3.2 kg SD 7.3) than for the people who we were unable to obtain measurements from at the extended follow-up (TDR −9.2 kg SD 10.6; UC −2.6 kg, SD 5.9). This suggests that the present results may reflect a more optimistic estimate of absolute weight loss in both groups.

We dealt with the missing data by using mixed models and extensive sensitivity analyses which consistently showed that the TDR group had greater weight loss than the nurse-delivered programme at 3 years. The trial was not powered to detect differences in cardiovascular risk factors. As such, the sample was insufficient to estimate the effect on blood pressure, and the confidence intervals range from no effect to a large effect. That said, the point estimate of the difference in blood pressure between arms is as would be expected from a prior meta-analysis, a reduction of ~3 mmHg, given the 3.6 kg difference in weight [19].

### Comparisons with other studies

A systematic review comparing TDRs with behavioural weight loss programmes reported that only four out of 12 studies followed people for 3 or more years [9]. A meta-analysis of these studies reporting longer-term follow-up showed a weight loss difference of only −1.3 kg (−2.9 to 0.2), somewhat less than the −3.3 kg (−5.2 to −1.5) seen here. Since this review, the DiRECT trial which tested a TDR programme for people with type 2 diabetes, has reported weight change at 2 years [20]. The rate of weight regain appears comparable to that we report here (Fig. S3). This is notable because the behavioural support in the DiRECT trial continued throughout the 2-year follow-up whereas in DROPLET it ceased after 6 months. Clarifying whether there is a benefit of ongoing support for the maintenance of weight-loss is important because it has implications for the cost of providing the intervention.

Weight regain in all trials of TDR is noticeably greater in absolute terms in the TDR programme than in the comparator group who lose less weight more slowly. This has prompted concerns that rapid weight loss is associated with rapid weight regain post-intervention. However, an experimental study which tested this hypothesis found no difference in the rate of weight regain following 15% weight loss achieved either with a TDR programme or a standard behavioural programme with more modest energy restriction over a longer period of time [21]. Instead it appears that the rate of weight regain reflects the absolute magnitude of weight loss, and our findings on weight regain are consistent with the extended follow up seen in trials of intensive weight loss interventions, which did not use a TDR approach, such as LookAHEAD [22]. Despite greater weight regain, the benefit of the greater initial weight loss with TDR persists to at least 3 years and a quarter of people who received a TDR programme were at least 10% lighter after 3 years, more than twice that in the UC group.

At follow-up, 7 in 10 participants in both groups reported currently trying to lose weight, similar to the proportion of people with obesity who report attempting to diet in the nationally representative Health Survey for England [23]. However in the trial, about half of the people who reported dieting were using a formal weight loss programme, which is associated with greater weight loss success than self-guided weight loss attempts. Our data suggest that people from more deprived neighbourhoods may have benefited relatively less at 3 years from the TDR than those from less deprived neighbourhoods, though the reasons for this are unclear. People from the most deprived neighbourhoods were no less likely to report being in formal weight loss programme at 3 years, but we cannot exclude the possibility that there were differences in attendance at weight-loss programmes between programme end and follow up especially since these programmes usually require self-funding [24, 25]. Overall these data suggest that the pattern of weight change reported here, and in most trials, of weight loss followed by weight regain, is likely to conceal a series of efforts by individuals at different times, with weight fluctuating on a shorter cycle time than that described by the mean weight change trajectory.

No previous trials of TDR programmes in routine care settings other than DiRECT have reported on metabolic or cardiovascular effects beyond 1 year. One previous experimental study that examined weight change 3 years after TDR also found that the rate of regain of body weight and body fat regain was greater in the TDR group compared with the control. However, as in the present study, the greater initial losses in the TDR group were such that there was a persistent advantage to the TDR intervention over control at 3 years [26]. In the present study, despite the greater weight loss following TDR, our study produced equivocal evidence on the longer-term benefits on blood pressure, but no evidence of greater improvements in glycaemic control or overall cardiovascular risk compared with UC at 3 years. These results are compatible with trials of other weight-loss interventions, which seem to suggest that partial weight regain removes more of the effect of weight loss on cardiometabolic parameters than might be expected simply from the change in weight [27]. This may reflect disproportionate changes in risk factors during periods of weight loss or gain.

### Implications of this research

This trial shows that offering a short TDR programme leads to long-term weight loss. We have previously reported that a TDR programme delivered by lay counsellors in the community is a cost effective treatment for weight loss [11]. The weight regain trajectories following TDR assumed in the cost effectiveness modelling were similar to those reported here, although weight regain in the UC group was somewhat slower than previously predicted such that the incremental cost effectiveness ratio would be lower. The DiRECT trial provides some evidence that offering a TDR improves glycaemic control in people with type 2 diabetes which is sufficient for at least a third of patients to be in remission at 2 years [20]. However, although underpowered to detect small effects, in the unselected population with obesity in DROPLET, there was little evidence that the TDR intervention led to greater improvements in cardiometabolic risk at 3 years. In practice it is these changes in risk factors for disease that are crucial to determining the cost effectiveness of interventions to treat obesity. Healthcare commissioners will need to carefully consider whether the routine provision of TDR programmes, which are substantially more costly than standard behavioural programmes, is warranted for people with obesity, but without pre-existing comorbidity.

## Conclusion

A short TDR programme shows long term sustained improvements in weight in a general population of people with obesity. However, the lack of additional benefits on cardiometabolic risk suggests that commissioning these services for the treatment of obesity in the general population may not prove cost effective.

## Supplementary information

Supplementary Material

## Data Availability

De-identified participant level data are available on request from the University of Oxford, Nuffield Department of Primary Care Data Access Committee for researchers who meet the criteria for access to confidential data: information.guardian@phc.ox.ac.uk.
