# Supplementary material for: Extended follow-up of a short total diet replacement programme: results of the Doctor Referral of Overweight People to Low Energy total diet replacement Treatment (DROPLET) randomised controlled trial at 3 years
Source: Int J Obes (Lond). 2021 Jul 23;45(11):2432–8. doi: 10.1038/s41366-021-00915-1 (PMC8528708; doi:10.1038/s41366-021-00915-1)
Supplement: Supplementary file 1 — Supplementary Material [file 41366_2021_915_MOESM1_ESM.pdf]

Table S1: Adjusted treatment effects for primary outcome under different missing data approaches

	**BOCF**		**LOCF**		**Multiple imputation**		**Completers only**	
	Usual Care (N = 138)	TDR (N=134)	Usual care (N= 138)	TDR (N=134)	Usual Care (N=138)	TDR (N= 134)	Usual Care (N=95)	TDR (N=104)
Unadjusted weight change from baseline*	-1.7 (6.1)	-4.5 (8.2)	-2.5 (6.7)	-6.7 (8.9)	-3.2 (8.3)	-6.1 (9.0)	-2.7 (7.7)	-6.3 (9.1)
Difference between groups †	-2.3 (-4.0, -0.6)		-3.9 (-5.5, -2.4)		-3.3 (-5.2, -1.5)		-3.3 (-5.2, -1.5)	
p-value	0.008		<0.001		<0.001		<0.001	

*Mean weight (kg) (SD)

† Adjusted mean difference (kg) (95% CI) using linear mixed effects model with fixed effects for randomisation group, baseline weight, visit and randomised group x visit interaction. Random effects accounting for practice and participant and within subject variance covariance matrix specified as unstructured. Age, gender and baseline type 2 diabetes status were included as covariates as these baseline variables were predictive of missingness

Table S2: Sensitivity analysis reporting adjusted treatment effects for primary outcome excluding self-reported weights

	**Self-reported weights excluded**	
	Usual Care (N=78)	Total Diet Replacement (N=90)
Unadjusted Mean Weight Change from baseline*	-2.5 (7.5)	-6.5 (9.3)
Difference between groups†	-3.6 (-5.5 to -1.7)	
p-value	<0.001	

* Mean weight (kg) (SD)

† Adjusted mean difference (kg) (95% CI) using linear mixed effects model with fixed effects for randomisation group, baseline weight, visit and randomised group x visit interaction. Random effects accounting for practice and participant and within subject variance covariance matrix specified as unstructured. Age, gender and baseline type 2 diabetes status were included as covariates as these baseline variables were predictive of missingness

Table S3: 3 year outcomes for participants with a baseline diagnosis of type 2 diabetes

	Change from baseline (mean ± SD)				Treatment difference	
	Total Diet Replacement	n	Usual Care	n	Adjusted difference (95% CI)	p value
Weight (kg)	-8.5 ± 10.1	19	-5.0 ± 5.1	17	-1.7 (-5.9,-2.4)	0.413
Fat mass (kg)	-6.2 ± 16.6	15	-3.8 ± 4.3	14	-1.7 (-7.0 -3.6)	0.526
Systolic Blood Pressure (mmHg)	-5.2 ± 21.2	14	-4.0 ± 13.4	13	-1.7 (-11.5; 8.1)	0.735
Diastolic Blood Pressure (mmHg)	-1.7 ± 9.9	14	1.6 ± 9.0	13	-3.0 (-9.5; -3.4)	0.356
HbA1c (mmol/mol)	6.2 ± 14.0	16	-2.1 ± 11.5	15	7.3 (-2.5; 17.1)	0.144
Oral diabetic medications	-0.31 ± 0.87	16	-0.25 ± 0.68	16	-	-
Waist circumference (cm)	-9.0 ± 11.6	15	-5.2 ± 7.5	14	-1.1 (-6.3; 4.1)	0.681
Fasting glucose (mmol/L)	0.1 ± 3.3	9	0.3 ± 2.8	9	-0.2 (-1.8; 1.5)	0.396
Fasting insulin (pmol/L)	-5.3 ± 22.8	8	-13.4 ± 31.1	7	0.3 (-26.0; -26.5)	0.983
HOMA- IR	-0.1 ± 0.4	8	-0.3 ± 0.7	7	-0.2 (-0.9;-0.4)	0.500
HOMA β (%)	-6.4± 28.7	8	-9.3 ± 24.9	7	-10.7 (-12.7; 34.2)	0.370
HOMA S (%)	1.3 ± 17.0	8	9.7 ± 18.9	7	1.4 (-29.3; 32.1)	0.928
Total Cholesterol (mmol/L)	0.1 ± 0.6	17	0.1 ± 0.5	16	0.1 (-0.4, 0.6)	0.669
HDL cholesterol (mmol/L)	0.09 ± 0.1	16	0.05 ± 0.2	16	0.1 (-0.23; 0.04)	0.171

LDL Cholesterol (mmol/L)	0.04 ± 0.55	12	-0.1 ± 0.42	14	0.2 (-0.3; 0.7)	0.392
Triglycerides (mmol/L)	0.1 ± 0.7	13	0.1 ± 0.7		-0.1 (-0.9; 0.7)	0.743

Table S4: 3 year outcomes for participants with a baseline diagnosis of hypertension

	Change from baseline (mean ± SD)				Treatment difference	
	Total Diet Replacement	n	Usual Care	n	Adjusted difference (95% CI)	p value
Weight (kg)	-4.0 ± 7.3	24	-4.7± 6.5	20	1.5 (-1.8,4.8)	0.374
Fat mass (kg)	-1.0 ± 11.3	17	-2.4 ± 6.4	19	2.1 (-1.9; 6.1)	0.309
Systolic Blood Pressure (mmHg)	-7.3 ± 18.0	19	-0.8 ± 13.7	18	-7.4 (-15.2; 0.5)	0.065
Diastolic Blood Pressure (mmHg)	0.3 ± 10.9	19	4.0 ± 11.2	18	-1.7 (-7.5; -4.1)	0.560
HbA1c (mmol/mol)	4.5 ± 12.5	20	-1.1 ± 9.8	18	3.7 (-2.1;9.6)	0.213
Antihypertensive medications	-0.21 ± 1.18	16	-0.05 ± 0.83	16	-	-
Waist circumference (cm)	-4.5 ± 7.6	18	-4.6 ± 8.7	19	0.6 (-3.3; 4.5)	0.765
Fasting glucose (mmol/L)	0.7 ± 3.3	15	0.1 ± 2.0	14	0.5 (-0.7; 1.8)	0.417
Fasting insulin (pmol/L)	-9.9 ± 39.9	14	-2.2 ± 57.7	13	-14.5 (-26.0; -15.5)	0.342
HOMA- IR	-0.2 ± 0.8	14	-0.1 ± 1.1	12	-0.36 (-1.1;-0.3)	0.316
HOMA β (%)	-15.7 ± 40.3	14	-0.3 ± 25.8	12	-9.6 (-35.7; 16.6)	0.474
HOMA S (%)	7.3 ± 37.8	14	20.4 ± 50.4	12	-13.1 (-38.6; 12.3)	0.312
Total Cholesterol (mmol/L)	-0.2 ± 0.9	20	-0.2 ± 0.5	18	0.1 (-0.4, 0.5)	0.835
HDL cholesterol (mmol/L)	0.00 ± 0.2	20	0.03 ± 0.2	18	-0.1 (-0.26; 0.01)	0.071

LDL Cholesterol (mmol/L)	-0.1 ± 0.57	17	-0.3 ± 0.5	16	0.2 (-0.2; 0.6)	0.422
Triglycerides (mmol/L)	-0.03 ± 0.6	18	0.1 ± 0.9	17	-0.01 (-0.5; 0.5)	0.974

Figure S1: Treatment effect by sub-group

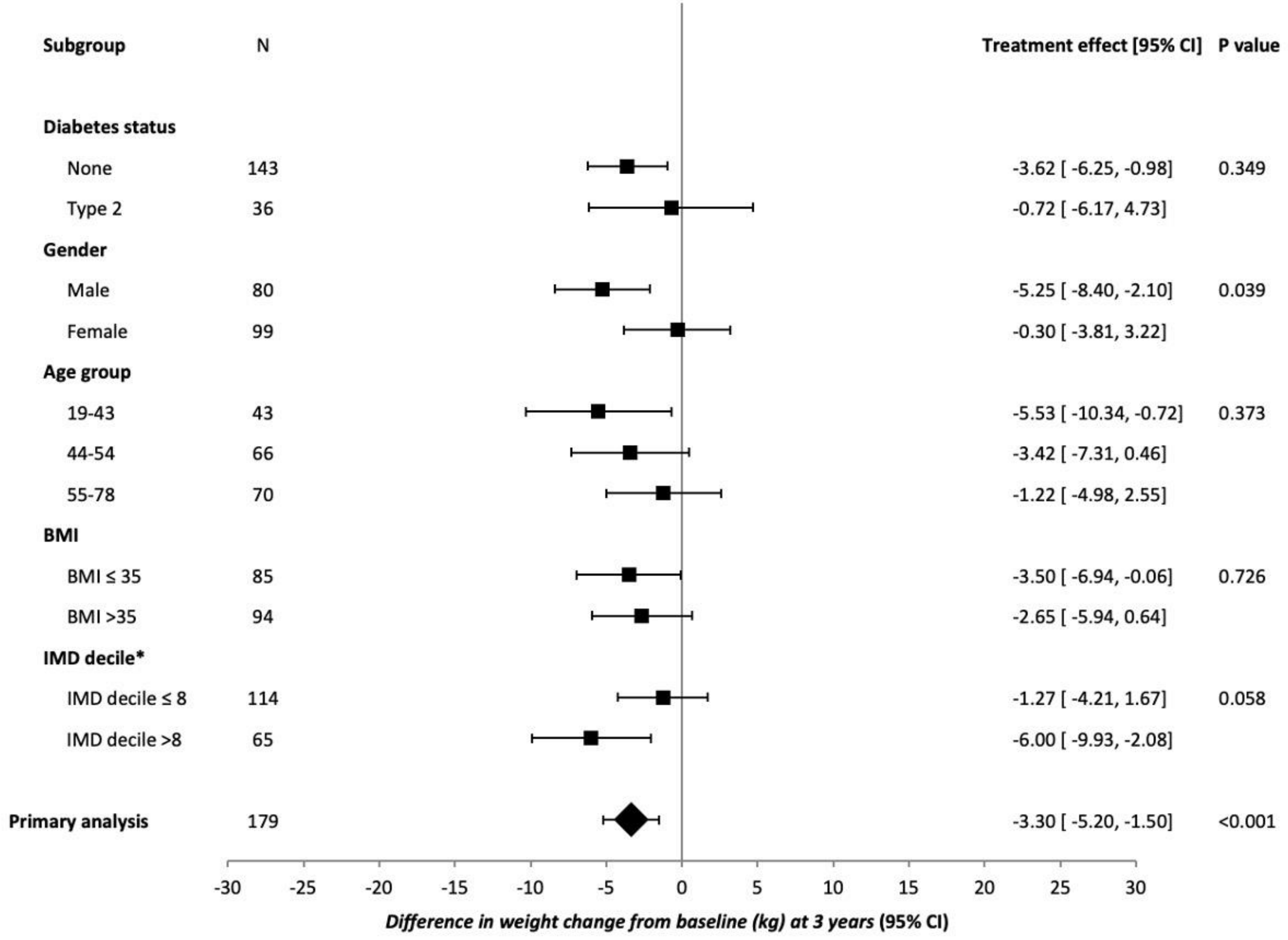

*IMD decile is an indicator of deprivation, with decile 1 being most deprived, and decile 10 the least deprived. IMD groups were compared using median split. IMD was not a pre-specified sub-group analysis, and was added after the statistical analysis plan was written, but before the primary analysis was conducted.

Figure S2:

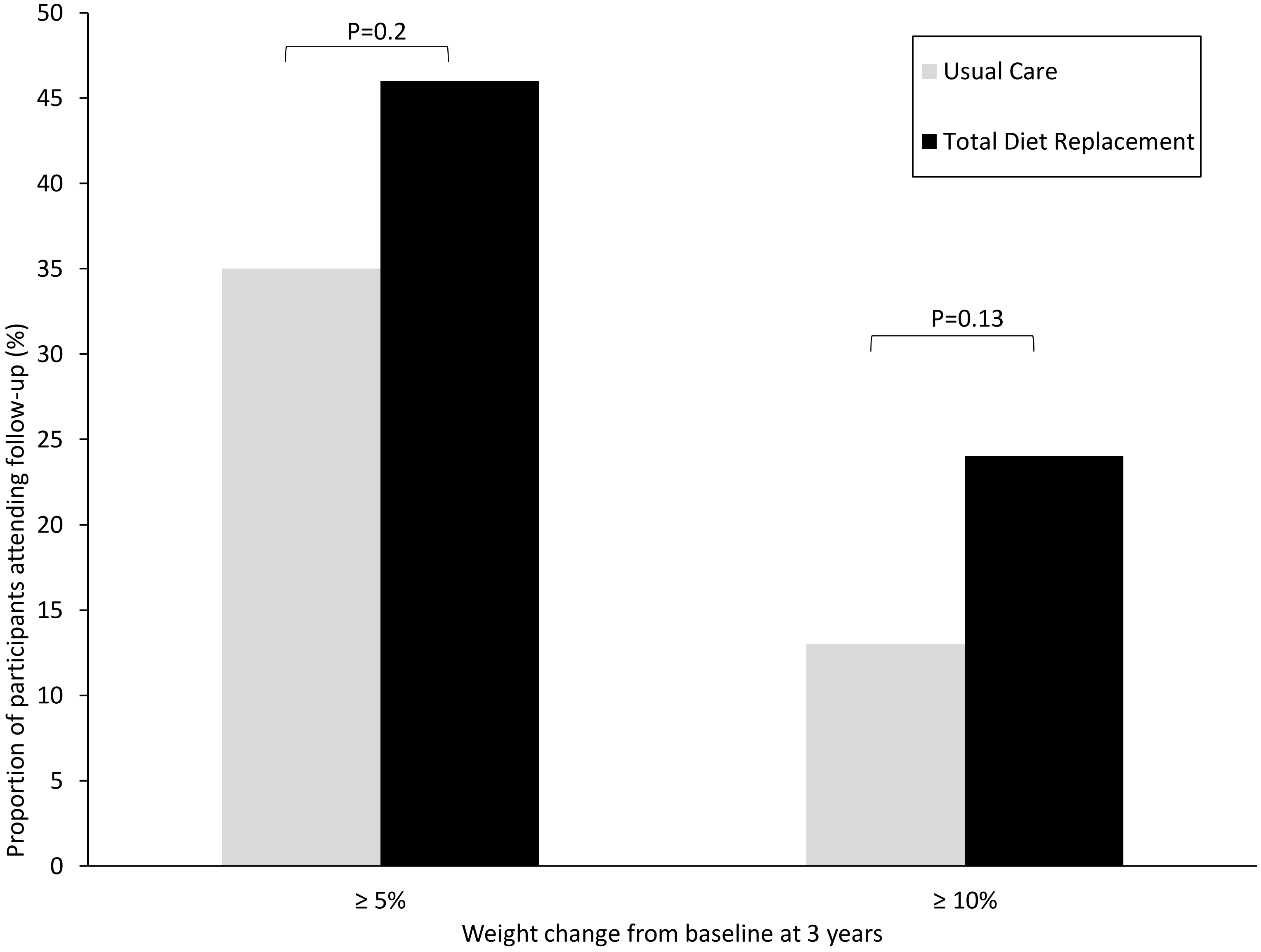

50
45
40
35
30
25
20
15
10
5
0
Proportion of participants attending follow-up (%)
P=0.2
P=0.13
Usual Care
Total Diet Replacement
≥ 5%
≥ 10%
Weight change from baseline at 3 years

Figure S3: Comparison of absolute weight change and estimated trajectories associated with TDR treatment in DROPLET and DiRECT clinical trials

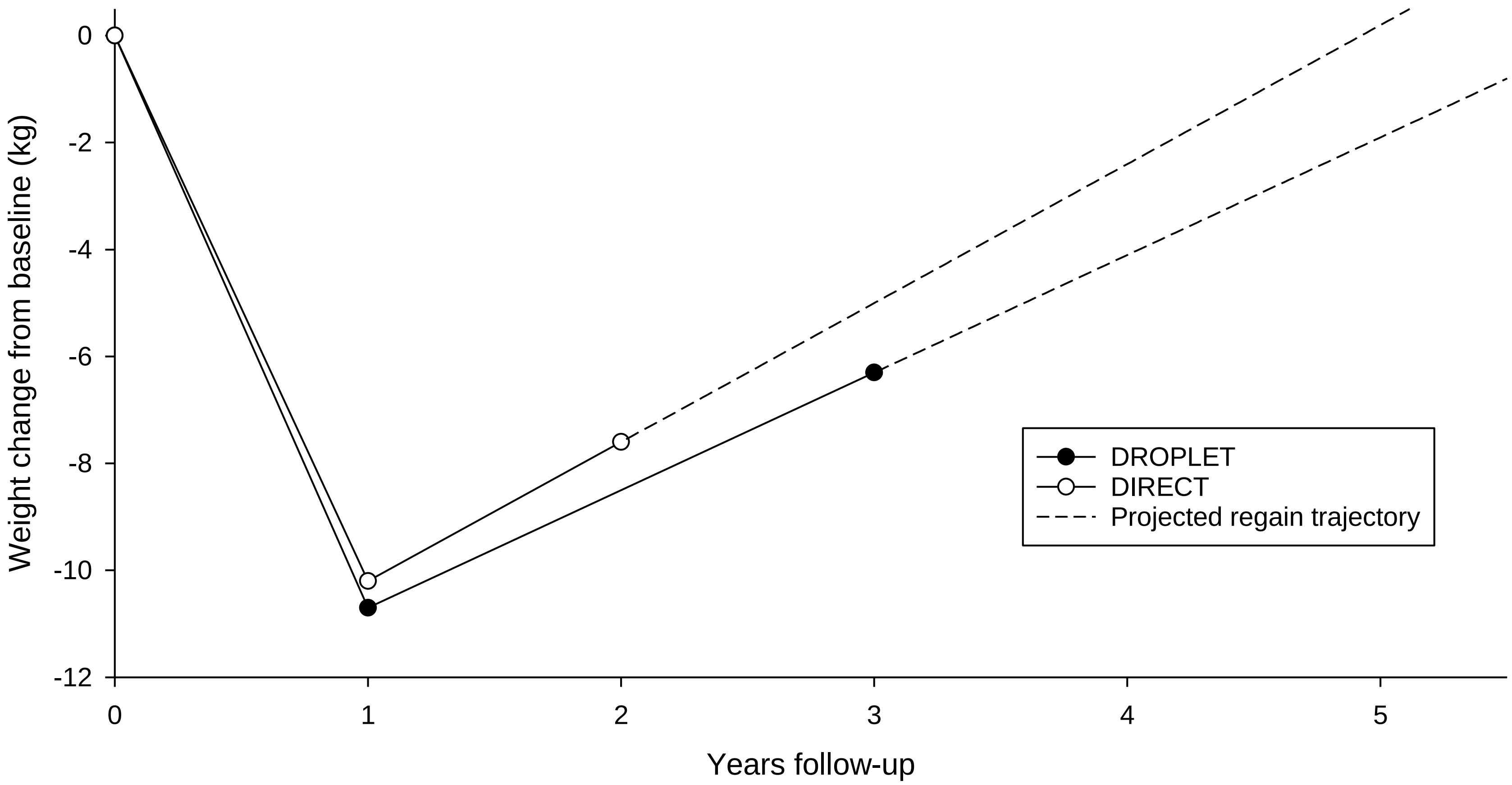

Weight regain trajectories based on observed data from the present study and that published by the 2 year follow-up of the DiRECT clinical trial participants.[25] The projected weight regain trajectories assume the weight regain trend after the last available measurement point is linear.
